# The translational roadmap of the gut models, focusing on gut-on-chip

**DOI:** 10.12688/openreseurope.13709.1

**Published:** 2021-06-04

**Authors:** Giulia Malaguarnera, Miriam Graute, Antoni Homs Corbera

**Affiliations:** 1 R&D department, Cherry Biotech SAS, Rennes, Brittany, 35000, France

**Keywords:** Gut-on-a-chip, Intestine-on-a-chip, Microbiota-on-a-chip, Colon-on-a-chip, Organ-on-a-chip, microfluidic, intestinal models

## Abstract

It is difficult to model *in vitro* the intestine when seeking to include crosstalk with the gut microbiota, immune and neuroendocrine systems. Here we present a roadmap of the current models to facilitate the choice in preclinical and translational research with a focus on gut-on-chip. These micro physiological systems (MPS) are microfluidic devices that recapitulate *in vitro* the physiology of the intestine. We reviewed the gut-on-chips that had been developed in academia and industries as single chip and that have three main purpose: replicate the intestinal physiology, the intestinal pathological features, and for pharmacological tests.

## Introduction

The human gastrointestinal (GI) tract primarily processes food and absorbs nutrients, water, and minerals, while also playing key roles in immunity and in different neuroendocrine processes ^1^. The physiological environments of different GI lumen sections are distinguished by their pH, redox potential, and transit time and they are deeply influenced by individual condition, diet, circadian clock, and physical activity ^2^. A healthy gut is marked by effective digestion and absorption of food, normal and stable intestinal microbiota, effective immune status, and general wellbeing ^3^. Poor quality diet, frequent use of antibiotics compromising gut microbiota biodiversity, aging and epigenetic factors have been associated with digestive diseases and linked to non-communicable diseases (NCDs) ^4– 6^. Dietary risk factors contribute to 11 million deaths and 255 million cases of morbidity worldwide, according to analysis of the Global Burden of Diseases (GBD) Study 2017 ^7^. In a more recent GBD report ^8^, the annualised rate of change between 2010 and 2019 for the Dietary risk factors assessed a decrease of -0.28, but an increase for the Metabolic risks factors (+1.46%), which can be also associated with the GI diseases ^9, 10^.

Considering the important role played by the gut in human physiology and pathology, considerable efforts have been invested to create relevant *in vitro* models for translational research and personalized medicine. Gut-on-chip (GOCs) models provide an advanced and unique approach to combine and preserve the original biological components, the biophysical architecture, and the biophysical phenomena of the gut *in vitro*. GOCs are organs-on-a-chip (OOC), small *in vitro* devices based on microfluidic technology that aim to replicate the minimal functional units of the intestine, enabling to culture intestinal cells and bioptic tissues ^11^. The GOCs have demonstrated so far capability to replicate: (1) specific physiopathological conditions (e.g. inflammation ^12^, intestinal bowel diseases – IBD ^13^, colon cancer ^14^); (2) *in vitro* drug pharmaco-kinetics (e.g. bioavailability assays ^15^, drug-to-drug interaction ^16^); (3) host-microbes interactions (HMI) ^17– 19^.

## Translational potentials and challenges of current gut models

**Figure 1.**
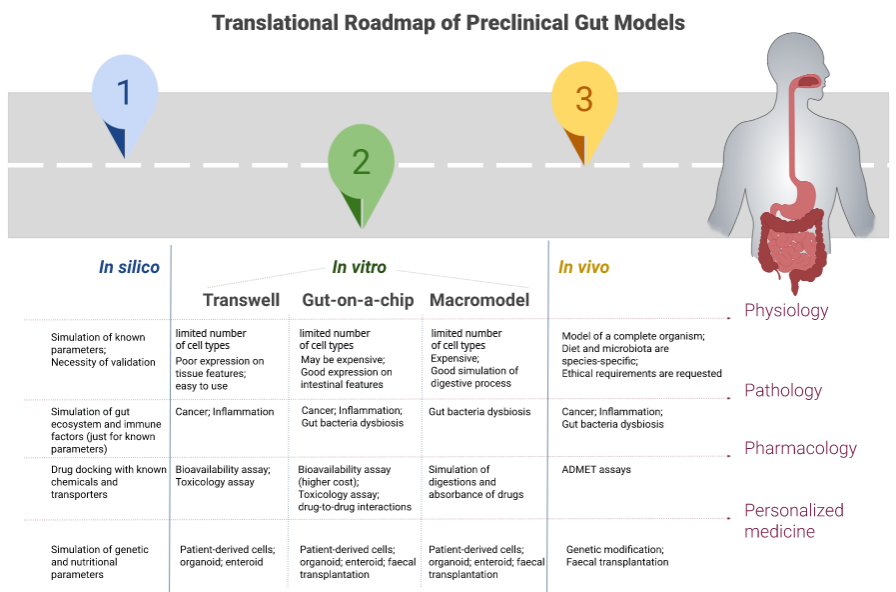
Roadmap of the translation in preclinical studies of gastrointestinal (GI) model in physiology, pharmacology, disease modelling and personalized medicine. ADMET=Absorption, Distribution, Metabolism, Elimination, and Toxicology.

The success rate of drug discovery and development from the preclinical phase and the clinical phases is about 32% ^20^. One of the main reasons of the high percentage of failures is due to the difficulty in finding preclinical models, both *in vitro* and *in vivo*, that resemble the human physiology, the pathological pathways, and the pharmacological response. Despite the disruptive therapeutic modalities such as gene therapy and immunotherapy, the development of more predictive *in vitro* model to study the treatment efficacy and toxicity is critical. In the preclinical research, the model roadmap to study the human GI tract pass by *in silico*, *in vitro*, and *in vivo* ( Figure 1). *In silico* approach is based in computer modelling and aims at producing algorithms or numerical models able to predict the drug effects. They have different level of complexity and include computational fluid dynamics (CFD) ^21^, ordinary differential equations (ODEs) ^22, 23^, aged-based modelling (ABM) ^24, 25^, and genome scale modelling (GSM) ^26^. The data acquired *in silico* requires validation to bridge the current gap between theoretical and experimental approaches ^27^. 

*In vitro* models commonly culture epithelial cell lines on Transwell® insert ^15^. The most used cell lines are the immortalized human-derived Caco-2, HT29 or HT29-MTX, or the animal-derived IPEC-J2. The advantages of culturing Caco-2 cells in Transwell ®, under static condition, are that: it is the regulatory standard model for drug bioavailability assays ^28^, it requires no ethical permissions as cells are commercially available, and it mimics features of both small and large intestine, despite being cells derived by colon cancer. However, there are three main limitations to this static Caco-2 *in vitro* model: (i) the human intestinal epithelium contains more than one cell type (enterocytes); (ii) mucus and flow are lacking; (iii) it is hard to accurately predict the human response to pathogens and drugs, as it does not include factors like nutrients, microbiota, hormonal factors, plasma carrier proteins, peristalsis speed, or bile acids ^29^. To overcome these limits, Caco-2 cells have been incorporated into macromodels and GOCs. 

Macromodels are bioreactors consisting of a series of compartments with different pressures, pH, flow rates, temperatures, and cells aiming to simulate the different GI sections by replicating their biochemical and biophysical parameters ^30, 31^. In these models, it is possible to evaluate the bioavailability of drugs and food, and their fermentation by using patient-derived microbiota ^32^. However, macromodels require costly lab equipment and space, need stabilization of the microbiota before use, and some of these systems do not mimic peristalsis and lack dialysis for removing microbial acid products ^27^. 

On the other hand, when Caco-2 are cultivated in alternative GOC models, they express the morphological and functional characteristic of the static *in vitro* Caco-2 monolayer, both in dynamic fluidic systems with transwells and simpler GOC ^33^. These models have the advantage to control intestinal histogenesis, physiology, mucus production, drugs, and nutrients response. This is possible by modulating several parameters: directional flow rates, mechanical deformation, fluid shear stress, and asymmetric stimulation of the apical and basolateral sides of developing epithelium. Delon *et al.* used a Hele-Shaw cell to investigate the main features of Caco-2 cells in a microfluidic device by applying several fluid shear stresses ^34^. They demonstrated that Caco-2 reach confluency within 5 days (earlier than in the static *in vitro* models) and that shear stress contributes to morphology, phenotype, and function of the epithelial layer. This turned into better mimicking tight junction expression, mitochondrial activity, mucus production, microvilli density, vacuolization, and cytochrome P450 (CYP450) expression. Gene expression study of Caco-2 on GOC revealed that expression of MUC17, a transmembrane mucin, was highly enhanced in the 3D villi model compared to a static monolayer culture ^34^. In a more recent study, the altered gene expression profile of Caco-2 was compared in static condition versus the flow culturing condition in a GOC after 21 days. Differences had been spotted in the cellular homeostasis, signal transduction, cell life cycle, and in the immunological responses ^35^.

Besides the translational advancement of these GOC models, there is still a lack of standardization among labs and intrinsic difficulties to scale-up their production. Moreover, like the other aforementioned *in vitro* models, the currently proposed GOCs are a simplified representation of the physiological system, including a reduced number of cell lines, and they generally do not comprise neuroendocrine or immune parameters. Interestingly, some GOC incorporate organoids, enteroids, and biopsies ^11, 36, 37^.

Another commonly used *in vitro* model of the gut consist of 3D organoids or enteroids, which can be grown from adult intestinal stem cells (ISCs), induced pluripotent stem cells (iPSCs) and primary intestinal epithelial cells (IEC). An advantage of these models is the reproduction of complex structures, including both epithelia and mesenchyme ^38^. However, 3D-organoids have lower success in modelling diseases such as IBD because of difficulties maintaining the quality and quantity of cells due to high occurrence of inflammation and pre-apoptosis. Challenges include viability (up to 48h), cost, and difficulties in accessing the lumen of the spheric structure for the application of microbiota and drugs. In pharmacology, there is potential to culture 2D organoids/enteroids in a monolayer to study drug interactions. Also in this case, when biopsies, enteroids or organoids have been integrated in a GOC it was possible to find some advantages in terms of better reproducibility of intestinal cytoarchitecture from a single donor ^11, 39– 41^, more reliability in the results for personalized therapy, or longer time in culture in the case of the biopsies ^37, 42, 43^.

In preclinical studies, a range of animal models are used, from small animals (mice and rats) to large animals (pigs, dogs, and non-human primates). This is done to study the effects of a potential treatment in a more complex system than the *in vitro* systems allow, considering the whole organism. Animal studies require ethical approval and their predictability is challenged by different diets and thus different gut microbiota composition from humans, different genomes, difficulties in handling and maintenance (particularly for large animals), and high costs ^27^.

## Focusing on GOCs: from academia to industries and their proof-of-concept

GOCs are microfluidic devices hosting cell or tissue cultures in a single chip. In Table 1, we list each chip, its main features, and the level of industry involvement. GOCs may be used for bioavailability assays, intestinal absorption of nutrients ^12^ and drugs, and real time evaluation of uptake and transports of drugs. The US Food and Drug Administration ( FDA), the European Medicine Agency ( EMA), and the World Health Organization ( WHO) recommend Caco-2 intestinal permeability assays as the standard model to determine the intestinal permeability rate and ratio of active pharmaceutical ingredients (API). These studies permit to compare the drug permeability from the apical to the basolateral side by considering the involvement of efflux transporter and active uptake transporters ( EMA Guideline on the investigation of drug interaction). Multiple transporters of the adenosine triphosphate (ATP) binding cassette (ABC) active transporter family such as P-glycoprotein (P-gp) or multidrug resistance protein- (MDRP1 or ABCB1) and multidrug resistance protein-2 (MRP-2 or ABCC2) efflux pumps are expressed by Caco-2 ^51^. A pharmaceutical compound needs to exhibit an apparent permeability (Papp) coefficient of > 90% compared with metoprolol, the gold standard for positive control in Caco-2 cells to be considered for exemption from bioequivalence studies; according to the Biopharmaceutical Classification System (BCS) ^52^. A systematic approach for the comparison of the BCS in static and in dynamic conditions on a GOC was done by Kulthong *et al.* ^15^, but no significant improvements were found in drug bioavailability, probably due to the very low shear stress applied in the GOC. In fact, in another GOC model based on 12-wells transwell insert connected to a bioreactor (Quasi-Vivo Kirkstall Ltd), applied fluid mechanical forces enhanced the absorbance of the fluorescein in a time-dependent manner ^48^. Comparing a thiol-ene GOC with static *in vitro* culture ^33^, the permeabilities of mannitol, insulin, and fluorescein isothiocyanate were not significantly higher. However, the Caco-2 grew and differentiated faster in the thiol-ene GOC, expressing P-glycoprotein 1 (P-gp), aminopeptidase activity and mucous proteins, which play important roles in the oral bioavailability. A GOC with integrated optical fibers developed by Kimura enabled to observe the transport of rhodamine 123 in real time ^47^. Two organoid-derived intestine-on-chip used the Emulate commercially available chip, also containing a polydimethylsiloxane (PDMS) membrane, for a small intestine-on-chip ^16^ and colon-on-chip ^11^ models. The advantage of using organoids derived from healthy donors compared to the Caco-2 model is that they better reproduce the intestinal cytoarchitecture, cell-cell interactions, transporters, and the expression of the CYP3A4. This is particularly relevant in studies on pharmacokinetics and pharmacodynamics. Duodenal epithelial cells are cultivated on top of the membrane, while human intestinal microvascular endothelial cells (HIMECs) grown at the bottom. Sontheimer-Phelps *et al.* have isolated human donor crypts, growing organoids, dissociating the spheres, and seeding the cell mixture onto the chip ^11^. This method replicated the mucus bilayer of the colon to a full diameter of 0.6mm. Unfortunately, they did not report how this affected the fluid velocity of the apical channel (height: 1.0 mm), nor did they take this into consideration when reporting the effect of shear on villi bending.

**Table 1.** List of main of gut-on-chip (GOC) models and their characteristics, including those developed in academia, in industries or in collaboration. AOI=Anoxic-oxic interfase; COC=Cyclic Olefin Copolymer; GOC=gut-on-chip; HMI=Host Microbes Interaction; IBD=Intestinal Bowel Disease; IOC=Intestine-on-chip; PC=Polycarbonate; PDMS=Polydimethylsiloxane; PE=Polyester; PET=Polyethylene terephthalate; PMI-CHIP=physiodynamic mucosal interfase-on-a-chip; PS=Polystyrene. Caco-2, CCD-18Co, CRC, and HCT-116 are colon cancer cell lines; HCoMEC=Human Colonic Microvascular Endothelial Cells; HIMECs=Human Intestinal Microvascular Endothelial Cells; HUVEC=Human umbilical vein endothelial cells; iPSC=Induced pluripotent stem cells; PBMC=Peripheral blood mononuclear cell; U937=human lung lymphoblast.

MODEL OF GOC	APPLICATION	CELLS/TISSUES	MEMBRANE (Y/N)	BULK MATERIALS	FLOW RATE (µL/MIN)	ACADEMIA (Y/N)	INDUSTRY (Y/N)
**HUMIX** ^18, 44^	- HMI - Disease modelling (colorectal cancer) - Pharmacology (pre- and probiotics)	Caco-2+ CCD- 18Co; primary CRC cells (T6)	Yes PC 1 µm pores	PC and silicone gaskets	25	Yes	No
**GOC** ^17, 45^	- Physiological characterization - HMI	Caco-2 + HIMECs	Yes PDMS	PDMS	0.5	Yes	Modified from Emulate
**AOI** ^19^	- HMI	Caco-2 + HIMECs	Yes PDMS	PDMS	0.833-3.333	Yes	Modified from Emulate
**IOC** ^11, 16^	- Physiological characterization - HMI - Drug-to-drug interaction	Primary, human derived organoids + HIMECs	Yes PDMS	PDMS	1	Yes	Yes Emulate
**PMI-CHIP** ^46^	- HMI - Disease modelling (IBD)	Caco-2 or patients’ organoids	Yes PDMS	PDMS	0.833-1667	Yes	No
**INTESTINAL** **MICROFLUIDIC MODEL** ^47^	- Pharmaceutical testing	Caco-2	Yes PE	PDMS and PE	N/A	Yes	No
**TUMOR-ON-A-CHIP** ^14^	- Disease modelling (Colorectal Cancer) - Pharmaceutical testing	HCT-116 + HCoMECs	No	PDMS	0.133	Yes	No
**GOFLOWCHIP** ^36^	- Physiological characterization	iPSC derived organoids	No	matrigel, clear cast acrylic plastic, silicone gasket, borosilicate glass	0.083	Yes	Yes
**ORGANOTYPIC-ON-A-CHIP** ^43^	- Physiological characterization - HMI	Biopsy (mouse intestinal section)	No	PDMS, collagen gel matrix	16.67	Yes	No
**DUAL FLOW BIOREACTOR** ^48^	- Physiological characterization - Pharmaceutical testing	Caco-2	Yes PC	PDMS	100-400	Yes	Yes Kirkstall
**USSING CHAMBER ON A** **CHIP** ^37^	- Disease modelling (IBD)	Human Intestinal Biopsy	Yes PDMS	Glass, petroleum jelly	4	Yes	No
**MOTIF** ^49^	- Physiological characterization - HMI	Caco-2, HUVECs, PBMCs, primary macrophages	Yes PET	COC	25-50	Yes	Yes ChipShop GmbH
**THIOL-ENE BASED** ** MICROFLUIDIC CHIP FOR** ** INTESTINAL TRANSPORT ** **STUDIES** ^33^	- Physiological characterization - Pharmaceutical testing	Caco-2	Yes Thiol-ene coated Teflon	PMMA, PDMS, tetra-thiol moieties	0.5-3	Yes	No
**GOC** ^15^	- Physiological characterization - Pharmaceutical testing	Caco-2	Yes PET	Glass, PET	0.4167	Yes	Yes Micronit
**NUTRICHIP** ^12^	- Physiological characterization - Disease modelling (inflammation)	Caco-2 + U937	Yes PET	PMMA, PS, PDMS	0.6-2	Yes	No
**ORGANOPLATE** ^13, 50^	- Physiological characterization - Disease modelling (IBD) - Pharmaceutical testing	Caco-2	No	PS, glass, proprietary polymers	N/A	No	Yes Mimetas

Several GOCs aim to target a specific disease, as in the case of the tumor-on-a-chip for nanoparticles developed by Carvalho and colleagues ^14^. Shear stress on HCT-116 cells (a human colon cancer cell line) and human colonic microvascular endothelial cells (HCoMECs) recreated the angiogenesis sprouting typical of colon cancer. To replicate the intestinal tubules, Beaurivage C *et al.* integrated extracellular matrix (ECM)-supported intestinal tubules grown from Caco-2 cells into their perfused microfluidic devices, OrganoPlate® ^13^. In this device, the cells exhibit cellular polarization, tight junction formation, and express key receptors. This GOC is easy to handle and allows different experimental settings for physiological, pathological, and pharmacological studies. However, limitations of this model are 1) the use of a rocker that, by switching inclination of +/- 7 degree every 8 minutes, results in non-uniform bidirectional shear stress; 2) the Caco-2 tubular structure of the chip remain stable only for 6 - 8h of perfusion ^50^.

Dawson and colleagues developed their dual-flow biopsy-holding chamber as an improved Ussing chamber ^37^. Biopsy culture was maintained for 68h at which point 80% of the tissue was alive as shown with lactate dehydrogenase (LDH) activity upon cell lysis. The longest culture time of intestinal explant tissue in a microfluidic device was reported by Baydoun and colleagues ^42^. In their study on a PDMS GOC, they demonstrated 3 of 9 biopsies to be intact upon histological observation after 8 days. Yissachar and colleagues implemented a gut organ culture, accommodating a mice gut tissue fragment in a bath of nutrients ^43^. The researchers cocultured *ex vivo* intestinal tissue with intestinal microbiota and investigated crosstalk with the immune system and expression of neuronal-specific genes. Limits of this model include the short length of experiments (structure degradation after 30–40 hours) and the microbiota overgrowth (24 hours). Scientists from Paul Wilmes group have developed and patented HuMiX, the “Human Microbial Cross-talk” model ^44^. This GOC co-cultures Caco-2 and bacteria, either *Lactobacillus rhamnosus GG* (LGG) or *Bacteroides caccae* ^18^. HuMiX is made from polycarbonate (PC) and therefore has the potential of large-scale production. However, the Caco-2 and the microbiota are separated by a PC membrane which may be a limitation, because only indirect interactions can be assessed. Furthermore, the rigid membrane does not allow the chip to simulate peristalsis. On the contrary, the peristalsis is part of the GOC described by Jalili-Firoozinezhad and colleagues ^17, 53^. This GOC is a Polydimethylsiloxane (PDMS) microfluidic two-channel device containing a porous membrane coated with ECM. The Caco-2 cells are cultured on top of the membrane, while below the human intestinal microvascular endothelial cells (HIMECs) lies. The peristaltic movement is controlled by two lateral vacuum chambers that stretch the membrane and regulate the suction force ^45^, like in the Emulate chips. The gut microbiota in the chip lived for up to 5 days, more than doubling the 48h of static Caco-2 monoculture. A modified chip, called anoxic-oxic interface-on-a-chip (AOI Chip) ^19^, was made by co-cultivating the Caco-2 cells with two obligate anaerobic bacteria, *Bifidobacterium adolescentis* and *Eubacterium hallii*. The authors demonstrated that AOI does not compromise the viability, mucin production, barrier function, and the expression of proteins in the intestinal epithelial layer. Moreover, to produce the anoxic environment in the chip while oxic culture media was flowed in the oxic chamber, it was sufficient to precondition culture media in an anoxic chamber. The same research group have more recently developed their own GOC called 3D physiodynamic mucosal interface-on-a-chip (PMI Chip) ^46^. The novelty introduced with the PMI Chip is the multiaxial stretching motion that provides the tortuosity of hydrodynamic flow with approximately 5% in cell strain at 0.15 Hz frequency. MOTiF biochips, designed by microfluidic ChipShop GmbH, is a microfluidic chip in polystyrol (PS) initially used to seed endothelial cells, human umbilical vein endothelial cells (HUVEC) ^49^. A limitation of the study is the oxygen gradient, which is difficult to measure or control, because bacteria and fungi are sensitive to the gas composition, temperature, and humidity ^54^. Following the differentiation of Caco-2 cells (which was faster compared to the transwell model), the authors demonstrated the possibility of colonization with bacteria ( *L. rhamnosus*) and the fungal pathogen candida albicans showing the competitive mechanism *in vitro*.

## Bulk and membrane materials

Materials employed in fabrication represent a crucial step and choosing a right material based on the application of the chip is not straightforward ^55^. One of the main bottlenecks to scale up the GOC are the materials used to manufacture them ^56^. PDMS is easy to prototype, elastic and optically transparent, but the costs are higher for mass production, it absorbs little hydrophobic molecules, such as drug compounds, it is permeable to carbon dioxide (CO _2_) and it has rather unstable surface properties ^57^. However, limited gas permeability of PDMS has been turned into an advantage in HMI studies, controlling for oxygen and anoxic flows to grow different species of gut bacteria ^19^. Thermoplastic materials, such as polycarbonate (PC), Poly(methyl methacrylate) PMMA, or Cycloolefins such as cyclic olefin polymers (COP) and copolymers (COC) are easier to produce in larger scale, through injection molding strategies ^58^. However, they need to be accurately selected to facilitate sterilization and the needed optical properties for a given assay. PC is easier to produce in larger scale, through injection molding strategies, and can be sterilized in autoclave, but it is more rigid, limiting its use to induce peristaltic deformations, and it has a poor resistance to organic solvents as well as some autofluorescence and sensitivity to ultraviolet (UV) radiation which could be minor inconveniences. COP and COC show low molecules absorption, minimum autofluorescence and excellent optical properties. However, thermoplastics are generally rigid materials and a flexible membrane, or a suitable biological structure should also be provided to induce realistic peristaltic deformations when needed in some GOC models ^59^. In most of the GOCs reviewed, membranes serve as support for cell culture (Caco-2 or primary cells) and to simulate peristalsis in combination with flow. They vary not only in manufacturing process and material, but also with regards to pore size, cell-to-cell distance, and overall porosity. Membrane permeability, a function of porosity, pore sizes and specific material properties like charge, is highly relevant for pharmacodynamic testing, such as bioavailability tests conducted in GOCs and other in vitro models. All of these GOCs have been trialed with synthetic membranes such as nylon, PDMS, PC, or polyester such as polyethylene terepthalate (PET). Some, for example Esch *et al.* ^60^ and Kim *et al.* ^53^ precondition or coat these membranes with collagen 1 to promote cell adhesion. Several papers lacked detail on the exact characteristics of the materials, simply stating that PC or PE from commercial transwells were used.

PC is one of the more commonly used synthetic membrane material due to low cost and rigid nature, as well as its resistance to autoclave pressure and temperature. Aspects such as thickness and porosity can be precisely controlled. However, it is not naturally biocompatible, leading some researchers to precondition the surface with collagen or mucin ^18, 61^. Other popular membrane materials are polyesters, including PET. Along with PC, they are widely established in transwell inserts and do not optically interfere in a critical way with microscopy.

Other bioengineering approaches for mimicking the villi structure had been explored and included in larger scaffolds (like the macromodels above described), but not in the GOC models. Other membranes that may be tested in GOC are a combination of synthetic and natural components ^62– 64^. Examples include 3D bioprinted membranes made of Poly(ethylene glycol) dimethacrylate (PEGDMA); gelatin methacrylate (GelMA); Lutrol; gelatin also mixed with chitosan; combination of fibrinogen, alginate, gelatin, and polyacrylamide; collagen; or silk proteins with spiral pattern ^27^.

## Conclusions and future perspective of GOC

GOCs are microfluidic devices that respond to the need of GI models that consider the ethical dilemmas involved in direct studies on humans (Declaration of Helsinki ^65^) and animal testing. In fact, The final aim of these devices is to refine, reduce, and ultimately replace animal testing based on the 3Rs’ ^66^ and utilise a closer model to the human physiology. Considering that the gut microbiota is also specie-specific and is influenced by nutrition ^67^, animal models are often less reliable models for GI compared to other organs ^68^. Efforts have been made to produce new GOCs or modify existing ones for new applications. There is however a lack of reported effort on stabilizing protocols to be applied on larger scales and ensuring the product is “fit for a purpose” ^69^. In fact, modifications to the geometry design and the protocols seems to be the major concerns of researchers in this field, making OOCs a niche not ready for a larger market and even less ready for the development and testing of therapeutic compounds. In the future, the GOCs described may have a higher output in *in vitro* studies on HMI, disease modelling, personalized medicine, and pharmacological studies. Fluid mechanical forces in GOCs enable to achieve intestinal physiological features more realistically when compared to other *in vitro* methods not incorporating biophysical stimulus ^70^. Therefore, GOCs can reduce the time for drug development and translational approaches with fewer ethical concerns than animal testing. The GOC approach is very promising but translation into industrial and commercial products aimed to cover the drug industry and healthcare markets require a larger effort to achieve robustness, to guarantee repeatability and to prove reliability.

## Data availability

No data are associated with this article.
