# Supplementary material for: The translational roadmap of the gut models, focusing on gut-on-chip
Source: Open Res Eur. 2023 Jan 18;1:62. Originally published 2021 Jun 4. [Version 2] doi: 10.12688/openreseurope.13709.2 (PMC10445823; doi:10.12688/openreseurope.13709.2)
Supplement: Peer review history

# Peer review: Reviewer response for version 2

**DOI:** 10.21956/openreseurope.16706.r30667

**Published:** 2023-02-02

**Authors:** J. J. Hickman, Mridu Malik

**Affiliations:** 1 Hybrid Systems Lab, NanoScience Technology Cente, University of Central Florida, Orlando, FL, USA; 2 Hesperos Inc, Orlando, USA

The authors have addressed all previous concerns.

Is the review written in accessible language?

Yes

Are all factual statements correct and adequately supported by citations?

Partly

Are the conclusions drawn appropriate in the context of the current research literature?

Yes

Is the topic of the review discussed comprehensively in the context of the current literature?

Partly

Reviewer Expertise:

I have a background in working with both static and dynamic in vitro organ models. I have worked on a static Caco-2/HT29-MTX cell based gut model to analyze the effects of engineered nanoparticles and other food additives on gut permeability, nutrient transport, and tight junction distribution in presence of a bacterial mock community. My experience with dynamic in vitro systems entails developing and optimizing a two-organ microphysiological system for secondary drug toxicity study. My work has made me well versed with the complexities involved in building a physiologically relevant microphysiological system and the significant factors that need to be considered to design it.

We confirm that we have read this submission and believe that we have an appropriate level of expertise to confirm that it is of an acceptable scientific standard.

# Peer review: Reviewer response for version 1

**DOI:** 10.21956/openreseurope.14783.r28371

**Published:** 2022-01-26

**Authors:** J. J. Hickman, Mridu Malik

**Affiliations:** 1 Hybrid Systems Lab, NanoScience Technology Cente, University of Central Florida, Orlando, FL, USA; 2 Hesperos Inc, Orlando, USA

The review article by Malaguarnera, Graute, and Corbera focuses on the current gut-on chip devices available in academia and the industrial sector, the challenges with using traditional static culture models, and the scale-up and design concerns with building the GOC models. The authors do a good job at pointing out the limitations to the different models included in the paper. The table listing the different GOC models available today is quite comprehensive, enabling the readers to have access to all the information in a single place. The article ends with a strong conclusion summarizing the current state of gut-on-chip research.

 I would suggest minor revisions to the paper. The article points out the limitation of Caco-2 models by stating they lack mucus and microbiota but fails to provide all relevant information on the available co-culture models of Caco-2/HT29 models with microbiota components. I would suggest expanding on this aspect more, and then outlining the challenges with maintaining the Caco-2/HT29/microbiota tri-culture for longer durations and its limited physiological relevance compared to GOCs.

 The placement of some information is erratic and lacks overall flow to the read. Changes have been suggested below to address these concerns.

**Introduction - Translational potentials and challenges of current gut models - Page 3:**

- Paragraph 1: The resource is for only academic drug discovery and development. 32% success rate is associated with only the pre-clinical phase. The same drugs are not necessarily going onto the clinical phase and succeeding. 
- Paragraph 2:GOCs are also *in vitro*, the difference arises between the flow of fluid across the intestinal barrier, static vs dynamic. Please make it clear that this paragraph deals with the static system.
- Include information on co-culture of Caco-2 and HT29 cells, where HT29 cells are mucin secreting cells, forming a mucus layer on the Caco-2 monolayer. Yes, there are limitations to the static gut model, however, complete lack of mucus is not one of them.
- See. Mahler G. J, Characterization of Caco-2 and HT29-MTX cocultures in an *in vitro* digestion/cell culture model used to predict iron bioavailability. 2009
- Recent studies have been working on including bacterial species, representing the gut microbiota, into an *in vitro* Caco-2/HT29 co-culture. The limitation to this is the restricted nutrient supply, and the time the mammalian and bacterial cells can co-exist in a static environment with build-up of bacterial metabolites and excessive growth rate of bacteria. Please address the same by referencing research on the topic.

**Page 4:**

- Paragraph 1: I wouldn't say GOCs are a simplified representation. There are a lot of complexities with constructing an organ-on-chip model. The better suited term would be 'more physiologically relevant' model.
- Inconsistency with the use of GOC and GOCs.
- Paragraph 2: I would add a reference paper to support this statement, “However, 3D-organoids have lower success in modelling diseases such as IBD because of difficulties maintaining the quality and quantity of cells due to high occurrence of inflammation and pre-apoptosis.”
- Paragraph 3: This paragraph should precede the introduction to different types of *in vitro* gut models in this section. The difference in data reproducibility when moving from animal trials to human trials is one of the primary reasons for the interest in the organ-on-chip model. So it is necessary to establish that before explaining the types of models.

**Page 7:**

- Right column, 10 lines from the bottom:  “… more than doubling the 48h of static Caco-2 monoculture.” - This is a contradiction to your claim in paragraph 2 of section 'Translational potentials and challenges of current gut models' that static 2D Caco-2 cultures lack microbiota.

**Page 8:**

- Left column, end of the initial paragraph: The statement- "… and the fungal pathogen candida albicans.." should be changed to "… and the fungal pathogen C *andida albicans* ...". *Candida albicans *should be in italics to correctly represent the scientific name of the organism.
- Left column, Bulk and Membrane paragraph: “PDMS is easy to prototype, elastic and optically transparent, but the costs are higher for mass production, it absorbs little hydrophobic molecules, such as drug compounds, it is permeable to carbon dioxide (CO2) and it has rather unstable surface properties ^57^.” - This statement seems to imply there is little absorption of hydrophobic molecules and that was not the authors intent.  To be more clear I think this should be worded "it absorbs low molecular weight hydrophobic molecules"

Is the review written in accessible language?

Yes

Are all factual statements correct and adequately supported by citations?

Partly

Are the conclusions drawn appropriate in the context of the current research literature?

Yes

Is the topic of the review discussed comprehensively in the context of the current literature?

Partly

Reviewer Expertise:

I have a background in working with both static and dynamic in vitro organ models. I have worked on a static Caco-2/HT29-MTX cell based gut model to analyze the effects of engineered nanoparticles and other food additives on gut permeability, nutrient transport, and tight junction distribution in presence of a bacterial mock community. My experience with dynamic in vitro systems entails developing and optimizing a two-organ microphysiological system for secondary drug toxicity study. My work has made me well versed with the complexities involved in building a physiologically relevant microphysiological system and the significant factors that need to be considered to design it.

We confirm that we have read this submission and believe that we have an appropriate level of expertise to confirm that it is of an acceptable scientific standard, however we have significant reservations, as outlined above.

## References

[1] : Characterization of Caco-2 and HT29-MTX cocultures in an in vitro digestion/cell culture model used to predict iron bioavailability. *J Nutr Biochem* .2009;20(7) : 10.1016/j.jnutbio.2008.05.006 494-502 10.1016/j.jnutbio.2008.05.006 18715773

# Peer review: Reviewer response for version 1

**DOI:** 10.21956/openreseurope.14783.r27228

**Published:** 2021-08-06

**Authors:** Rosalba Parenti, Nunzio Vicario

**Affiliations:** 1 Department of Biomedical and Biotechnological Sciences, Section of Physiology, University of Catania, Catania, Italy

In this manuscript entitled " *The translational roadmap of the gut models, focusing on gut-on-chip*", Malaguarnera, Graute and Corbera sought at reviewing current knowledge on experimental models of gastrointestinal diseases.

 We find this review well written and pleasant to read. It is also well organized and balanced. The critical vision of the authors is offering significant insights, rising interesting scenarios such as the one pointing on increasing the reliability of gut-on-chip modelling.

 The authors took into account the complex physiological environment of gastrointestinal lumen sections and advised on the reductionistic approach of gut-on-chip and organs-on-a-chip. The review is also clearly pointing out that gut-on-chip modelling is a valuable tool when studying specific physiopathological conditions, pharmaco-kinetics and host-microbes interactions.

 We do not have any major points to rise, and we believe that the manuscript is ready to be shared with scientific community and with colleagues in the field. We just have some minor points:

- Figure 1 describes a roadmap of translational study of gastrointestinal models. Authors clearly described most of the concepts herein described in the text. Perhaps, it might be useful to add a further brief description of in silico approaches to the manuscript. In particular on the simulation of known parameters, drug docking and genetic and nutritional parameters, highlighting the limitation of such an approach.
- The part of *in vivo* modeling also deserves to be expanded a bit. Indeed, *in vivo* studies on axes between gut and other organs, such as gut-to-brain axis, is today of critical importance and of great interest for the scientific community in the field. See Tan et al. Nature. 2020. PMID: 32322067 and Zimmerman et al. Nature. 2019. PMID: 30918408.

We believe that the manuscript is overall balanced, critical and it can be considered for indexing also if authors will decide not to include the previous minor points.

Is the review written in accessible language?

Yes

Are all factual statements correct and adequately supported by citations?

Yes

Are the conclusions drawn appropriate in the context of the current research literature?

Yes

Is the topic of the review discussed comprehensively in the context of the current literature?

Yes

Reviewer Expertise:

Analysis of the mechanisms underlying the processes of neurodegeneration and neuroinflammation and intercellular communication in homeostasis and disorders of the nervous system. Analysis of the mechanisms responsible for the transformation of the physiological phenotype into cancer as well as the application of advanced technologies aimed at the development of new diagnostic and therapeutic approaches to cancer.

We confirm that we have read this submission and believe that we have an appropriate level of expertise to confirm that it is of an acceptable scientific standard.

## References

[1] : The gut-brain axis mediates sugar preference. *Nature* .580(7804) : 10.1038/s41586-020-2199-7 511-516 10.1038/s41586-020-2199-7 32322067PMC7185044

[2] : A gut-to-brain signal of fluid osmolarity controls thirst satiation. *Nature* .568(7750) : 10.1038/s41586-019-1066-x 98-102 10.1038/s41586-019-1066-x 30918408PMC6483081
